# Investigation of the Properties of Anti-Friction Coatings Deposited with Different Casting Methods

**DOI:** 10.3390/ma17112662

**Published:** 2024-06-01

**Authors:** Tomas Kačinskas, Saulius Baskutis, Jolanta Baskutienė, Lina Kavaliauskienė

**Affiliations:** Department of Production Engineering, Faculty of Mechanical Engineering and Design, Kaunas University of Technology, Studentu St. 56, 51424 Kaunas, Lithuania; t.kacinskas@iremas.lt (T.K.); jbask@ktu.lt (J.B.); lina.kavaliauskiene@ktu.lt (L.K.)

**Keywords:** microstructure, tin-based Babbitt alloy, coating, clad welding, casting

## Abstract

This article presents the research results of depositing anti-friction coatings (Babbitt) using three different casting methods: static casting, flame soldering, and clad welding. Babbitt alloy coatings deposited with different casting methods are discussed and explained in terms of changes in the coating properties, such as the microstructure, hardness, strength, and chemical composition. The results showed significant differences in the aforementioned properties, depending on the chosen coating deposition method. The results of the tests confirmed the importance of using shielding gas during deposition to ensure the chemical composition of the coating. The analysis revealed that decreases in the amounts of antimony and copper in the Babbitt coating compared with the initial concentrations were influenced by selective evaporation, oxidation, and the coating process parameters associated with different coating methods. To maintain the desired balance of mechanical properties in Babbitt coatings, it is important to control the antimony and copper contents. Clad welding deposition using a non-consumable tungsten electrode and argon shielding gas achieved a chemically stable coating quite close to the initial chemical composition of the Babbitt alloy.

## 1. Introduction

In the field of metallurgy and resurfacing, the application of antifriction coatings has long attracted the attention of both research and industry. The present study explores the complex practical relationship between theory and application to reveal the structural properties of anti-friction coatings and their formation under various methods, with a comparative analysis used to determine the most effective method. Antifriction coatings, which are renowned for their versatility and utility in various industries, have also generated interest in part restoration. This interest is driving research, particularly in metallurgy and materials science.

This paper focuses on the antifriction coating Babbitt, also known as white metal, which is used in the manufacturing and restoration of specific parts. Tin-based Babbitt alloys, which are the objects of this research, are characterized as casting alloys with microstructures that usually appear in the three-phase form α, β, η, where α is the antimony (Sb) and copper (Cu) in the tin (Sn), which together form a soft and ductile matrix; β represents the angular SnSb phase crystals; and η represents the acicular precipitates of the Cu_6_Sn_5_ phase [1]. These coatings are commonly used as bearing materials in various devices and machines, such as turbines, engines, motors, compressors, and pumps [2,3,4,5,6]. Babbitt alloy coatings are applied to reduce friction between moving parts, thereby reducing wear on components in contact with each other and extending the service lives of devices and machines [7,8,9,10]. Babbitt’s good damping properties help reduce vibration and noise in rotating machinery. When applying Babbitt alloy coatings, various spraying technologies can be selected, including low-pressure cold spray technology, arc spray technology, flame spray technology, and thermal spray technology [11,12,13,14]. However, studies have shown that the interface between the coating and substrate is mechanically bonded in coatings fabricated via cold spraying or thermal spraying, which peel off at high temperatures or under relatively high loads [15]. The deposition efficiency during cold spraying depends, to a significant extent, on the speed of the particles in contact with the coated surface. The particle velocity should remain within the interval between the critical and erosion velocity, as a coating will not form when the particle velocity is lower than the critical velocity during impact due to insufficient bonding. However, if the impact velocity is too high and exceeds the erosion threshold, there is a high probability that the already-deposited layers will be removed [16]. In addition, it is difficult to avoid the formation of pores or oxidation in the coating using spray techniques [17,18,19,20]. For example, depending on the spray parameters, the porosity of the coatings when using the thermal spray method can reach up to 16% or higher by volume [21]. Laser coating technologies allow the fabrication of coatings with better functional parameters than those using thermal spraying technology [22,23]. However, laser technology requires that the coated surfaces be carefully prepared with a suitable structure while avoiding any contaminants, which is often difficult to implement in a manufacturing environment. In addition, the technological process is rather complicated, requiring expensive equipment and relatively high costs [24,25]. Other possible methods for fabricating Babbitt alloy coatings involve casting.

Modern research and development in casting technology contribute to the continuous improvement of Babbitt alloy coatings in solving engineering problems. When using coating casting methods, it is important to ensure the uniformity of the coating and high-quality adhesion to the base metal. Thus, special attention must be paid to the preparation of the surface to be coated, the chemical composition of the alloy, temperature control, and the casting technology itself [26,27,28]. Babbitt alloy coatings fabricated via casting cannot be strengthened via cold working. This factor is influenced by the relatively low recrystallization temperature [29]. Uneven heat input during casting affects the grain sizes of Babbitt alloys [30,31]. Therefore, the solidification rate affects the microstructure and hardness of the coating. Fine-grained structures created through controlled thermal processes improve mechanical strength and wear resistance, while proper thermal control reduces the presence of unwanted inclusions in the matrix [12]. Studies have shown that a slower cooling rate leads to the formation of larger SnSb and Cu_6_Sn_5_ precipitates and a coarser microstructure. Conversely, an increase in the solidification rate leads to a decrease in the size of the precipitates [15,32]. Therefore, during the Babbitt casting process, control of the cooling rate has a significant influence on the formation of the microstructure and its adaptation to specific performance requirements. The chemical composition of the antifriction coatings is also an important choice in the production or restoration of machine and equipment parts. In Babbitt alloy coatings, Sn is the main component that influences the properties and performance of the coating, providing a soft matrix for the other metals. Sn is characterized by its softness, ductility, and low coefficient of friction, enabling the coating to adapt to the irregularities of in-contact surfaces, thereby reducing friction and wear between moving parts [33,34,35,36]. In addition, Sn provides good adhesion of the coating to the base metal, extending the durability of the coated parts. Thus, a properly determined amount of Sn in the coating plays a vital role in influencing the tribological, mechanical, and chemical properties of the deposited layer [37,38]. Cu increases the thermal conductivity of the coating [26,39,40,41]. This property reduces the possibility of overheating among the contacting parts and maintains the temperature balance. Cu enhances the hydrophobic properties of the coating through its inherent corrosion resistance [42,43]. The protective oxide layers formed under the influence of Cu effectively protect both the coating itself and the base metal from rust. Cu helps prevent deformation and plastic flow of the coating under load, through which the required distance between the contacting surfaces is kept stable [44]. Sb adds hardness to the Babbitt alloy coating and increases its wear resistance [45,46,47,48]. This factor is critical in applications where the contacting surfaces experience high friction, such as in bearings or other sliding surfaces [14,49,50,51]. Another important aspect is that Sb promotes the formation of a fine-grained structure in the coating, which contributes to the mechanical strength, crack resistance, and wear resistance [52,53]. Studies have shown that precipitation of the SnSb phase has a decisive influence on the wear resistance of bearings covered with a Babbitt alloy coating [54]. On the other hand, research showed that if the amount of Sb in the alloy exceeds 18–20%, then the wear resistance will begin to decrease [55,56].

The aim of this study is to determine the characteristic properties of Babbitt alloy coatings and the consequences of creating such alloys using different casting methodologies. Firstly, we recognize that practical applications extend beyond the confines of the laboratory. Thus, this study includes a holistic study of Babbitt alloy coatings, including their characteristics, casting techniques, technical parameters, and applications in real manufacturing. We acknowledge the challenges of practical experimentation, as Babbitt alloy coatings can be fabricated using an oxidizing flame with burning gas (flame soldering) via melting in a furnace and pouring directly from a crucible onto the surface of the part (static casting) or with an electric arc created by a tungsten electrode in an inert gas environment (clad welding). We explore the intricacies of Babbitt alloy coatings, carefully examining their physical, mechanical, and metallurgical properties. The relationship between the importance of chemical composition, mechanical properties, and microstructure in the context of this research topic is also discussed. The goal is to reveal the nuanced differences when using different casting methods and ultimately provide sound recommendations for practical applications.

## 2. Materials and Experimental Details

**Figure 1:**
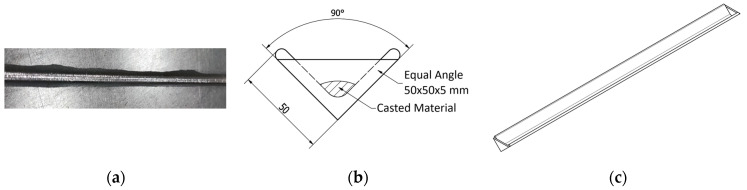
Casting of Babbitt rods: (**a**) cast rod; (**b**) mold cross-section; and (**c**) mold sketch.

The Babbitt casting process began by preparing the Babbitt material in the form of rods. The most vital element in this process was to maintain the original structure of the Babbitt material in the recast rods. The use of Babbitt rods (Figure 1) was based on examples of additional materials used in welding, such as electrodes and welding wires.

The Babbitt material was melted in a crucible in an electric heating furnace with automatic temperature control at a temperature of 370 ± 10 °C. During Babbitt heating, dehydrated ammonium chloride was used as a flux to clean the Babbitt of impurities and contaminants. Hot Babbitt oxidizes, and thus it was necessary to shorten the heating and casting time as much as possible when forming the rods. In addition, Babbitt requires vigorous stirring during heating to achieve a more uniform and finer grain structure. For this purpose, we used a special bell that moved along the bottom of the bath until gas bubbles ceased. The stirring speed and time were not automated. Instead, the relevant control parameter was continued agitation of the bath until the gas bubbles stopped spreading. After remelting the Babbitt alloy into rods, the chemical composition was determined (Table 1).

Forming the Babbitt material into rods not only facilitated the casting process but also ensured that the final Babbitt alloy coatings were of high-quality and had reliable properties.

Before coating with the Babbitt alloy, the surfaces underwent the degreasing, fluxing, and tinning operations applicable to all Babbitt casting methods. The quality of degreasing was assessed by wetting the degreased surface with water. The degreased surface was confirmed to be free from water film damage (grease marks). The surfaces were degreased again if smooth surfaces remained unwetted. Fluxing is a casting preparation step in which reagents such as saturated zinc chloride play a key role. This method effectively removes oxides and dirt from metal surfaces, ensuring that the surface to be coated is chemically active and receptive to the Babbitt material. This etching step is critical in promoting uniform and reliable adhesion between the metal substrate and the Babbitt alloy. Tinning is the last step in the preparation process before casting the Babbitt alloy. First, the surface of the specimen to be coated is heated to 270–300 °C. When the Sn starts to melt, it is immediately distributed over the entire surface in an even and thin layer. A properly applied layer of Sn should be a uniform dull silver color. Any other color, such as yellow, indicates that the tinning process was performed incorrectly and that the Sn is oxidized and unsuitable for a Babbitt alloy coating, as Babbitt will not adhere properly to such a surface.

**Figure 2:**
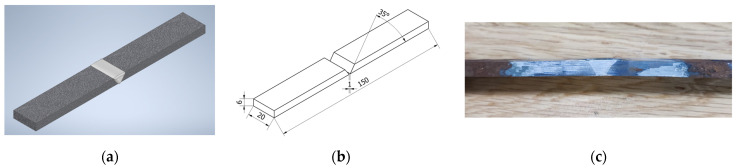
“V-shaped” specimen type: (**a**) specimen model; (**b**) sketch of the specimen; and (**c**) manufactured specimen.

Two different types of specimens were developed, each adapted to specific testing and evaluation processes. The first type of “V-shaped” specimen consisted of two metal plates carefully joined together in the same way as the welding specimens. These plates formed a V-shaped groove designed to accommodate the three different Babbitt depositing methods investigated (Figure 2).

**Figure 3:**
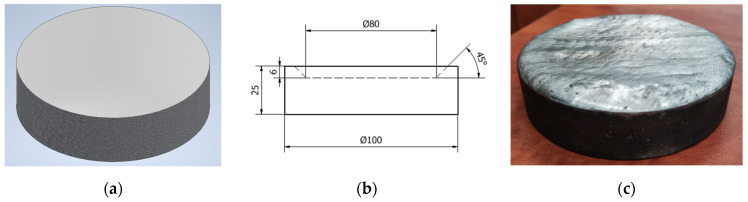
“O-shaped” specimen type: (**a**) specimen model; (**b**) sketch of the specimen; and (**c**) manufactured specimen.

This design choice is compatible with the objective of tensile testing the specimens to gain valuable insights into the structural integrity, bond strength, and mechanical properties of Babbitt coatings. Examples of “O-shaped” specimens instead featured a flat round configuration with side walls forming a bath (Figure 3). These specimens were created for a comprehensive evaluation that included hardness testing, chemical composition analysis, and microstructural analysis.

**Figure 4:**
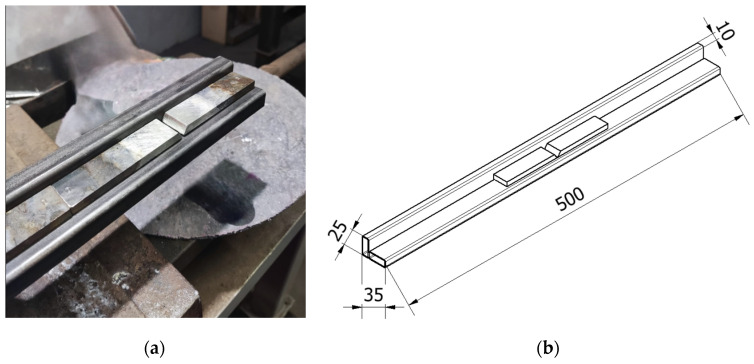
Stand for specimen preparation: (**a**) general view and (**b**) sketch of the stand.

Special stands were designed and manufactured for specimen preparation (Figure 4).

Using the static casting method, the Babbitt material was melted and refined in a furnace. The Babbitt coating was created by directly pouring liquid Babbitt from a crucible onto the prepared surface. To ensure the accuracy of the experiment, the temperature under the Babbitt coating was measured after pouring the coating onto the surface of the base metal (carbon steel S355J2 was used as the substrate). The temperature reached 378 °C, which was suitable to ensure that Sb, one of the main elements, was not burnt due to the introduction of excessive heat in Babbitt casting.

**Figure 5:**
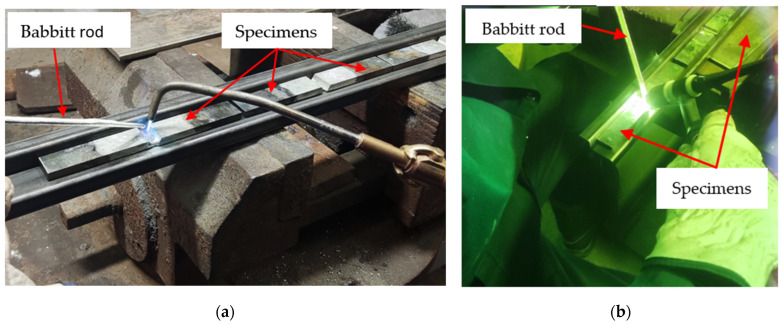
Babbitt alloy coating procedure: (**a**) coating using the flame soldering method and (**b**) coating using the clad welding method.

For the flame soldering method, Babbitt was melted using an oxidizing flame generated by a burning mixture of propane and oxygen gases at a ratio of 1:4 (Figure 5a). 

For the clad welding method, we used a digitally controlled electrode power source with resonant intelligence, a Fronius TransPocket 2500 TIG welding machine (Fronius International GmbH, Wels, Austria), a non-consumable Abicor Binzel 1.6 mm diameter WP green TIG tungsten electrode (Alexander Binzel Schweisstechnik GmbH & Co., KG, Buseck, Germany), 20 A of current, and argon shielding gas with a concentration of 99% (Figure 5b).

A PMI-Master PRO spectrometer (Oxford Instruments Analytical GmbH, Uedem, Germany) was used to determine the chemical composition of the Babbitt. A microstructural analysis of the coating was performed using a Zeiss Axioscope A1 microscope (Carl Zeiss Microscopy GmbH, Jena, Germany), Zeiss Axiocam 208 color imaging camera, and Zeiss Labscope 4.2 version software. The tensile tests on the specimens were carried out with a Tinius Olsen H10KT universal tensile test machine (Tinius Olsen Ltd., Salfords, Redhill, UK) featuring a load capacity of 10 kN at room temperature (22 ± 1 °C), with 50 ± 5% relative humidity at a test speed of 13.8 mm/min. Hardness measurements were carried out with a Mitutoyo HR-530 Series Hardness Testing Machine (Mitutoyo Corporation, Kanagawa, Japan) under a load of 10 N with a Ø5.0 mm indenter.

## 3. Results and Discussion

### 3.1. Chemical Composition

The chemical composition of Babbitt has a significant influence on the properties of the coating and its suitability for various applications. An analysis of this composition was thus necessary to ensure the composition and structural integrity of the material, especially in cases where the material may be exposed to heat. The results of this analysis are presented in Table 2. 

The results of the chemical analysis of the three Babbitt deposition methods revealed differences in the contents of the key elements, primarily Sb and Cu. In the clad welding method, the amounts of Sb and Cu were relatively higher at 13.10% and 5.57%, respectively. The flame soldering method yielded slightly lower amounts of Sb and Cu at 12.70% and 3.69%, respectively. The content of Sb in the Babbitt deposited during static casting was 12.40%, while that of Cu was 3.73%. These differences are attributable to several factors, including differences in temperature, exposure to oxygen, the duration of the coating process, and the evaporation rates during the deposition process. During deposition, the molten Babbitt was exposed to oxygen and other oxidizing agents which affected elements more reactive than Sn, such as Cu and Sb. The formed oxides then reduced the concentrations of Cu and Sb in the coatings obtained using the flame soldering and static casting methods. Under the clad welding method, the deposition process occurred in a protective argon gas environment, which eliminated the formation of oxides. Consequently, the amounts of Cu and Sb in the deposited layer remained practically unchanged. We also recorded the duration of the experimental coating process. Studies have shown that the static casting deposition method requires the most time. Compared with the time investment under the static casting method, the duration of the open-flame soldering method was reduced by about 60%, while that of the clad welding method was reduced by 65%. As a result, less Cu and Sb was vaporized under the clad welding method.

Because a more homogeneous structure forms under the clad welding method, the amount of Sb was higher due to Sb separating more evenly and merging into the common matrix during deposition. Conversely, with the flame brazing and direct casting methods, the amounts of Sb and Cu were slightly lower due to uneven heat exposure during the formation of solid precipitates of the aforementioned elements.

### 3.2. Tensile Test

**Figure 6:**
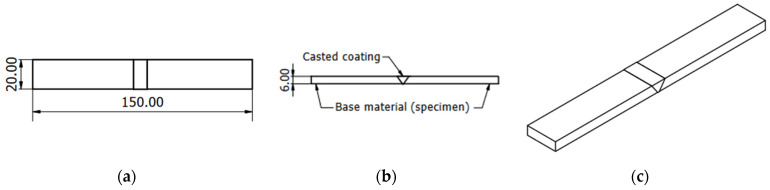
Tensile test specimen: (**a**) top view; (**b**) side view; and (**c**) general (isometric) view.

Tensile tests were performed to determine the ultimate tensile strength of Babbitt alloy coatings fabricated using the three different methods under static loading, according to the standard EN ISO 4136:2022 [57]. In total, 15 specimens were prepared for tensile tests. The specimens for tensile measurements were rectangular in shape, with dimensions of 150 mm × 20 mm × 6 mm (Figure 6). During the tests, the relationship between the force and the tensile speed was determined experimentally. Calculations showed that the selected test speed was 13.8 mm/min. 

**Figure 7:**
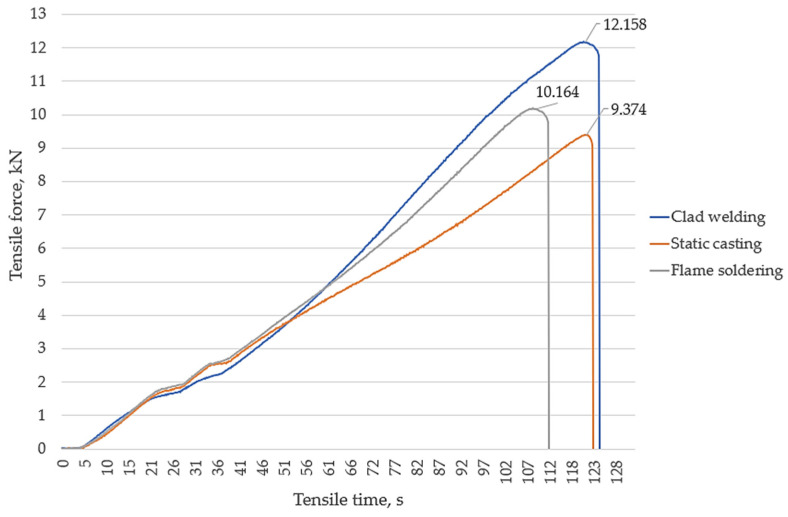
Tensile curves of the specimens.

Detailed results of the tensile strength testing experiment are presented in Table 3, and the tensile curves are presented in Figure 7. In these curves, we selected the specimens that withstood the highest loads during the test. The maximum load force and strength values were determined according to the instructions of the standard ISO 6892-1:2019 [58].

The testing machine recorded the force (stress) and length of time which the specimen could withstand the load before breaking.

The calculated averages showed that the ultimate tensile strength limit of the coatings for the specimens obtained was 59 MPa via static casting, 71 MPa using the flame soldering method, and 83 MPa using the clad welding method. These results demonstrate that the coating fabricated with the clad welding method had the highest ultimate tensile strength of all three methods, primarily due to the higher amount of Sb in the coating. Sb formed solid solutions with Sn in the Babbitt coating because the Sb atoms occupied interstitial space, replaced Sn atoms in the crystal lattice, and formed intermetallic compounds with Sn [59]. As the amount of Sb in the alloy increased, more intermetallic compounds formed, which increased the general strengthening of the alloy and contributed to its tensile strength. In addition, the Sb yielded a Babbitt alloy with a finer grain structure. Finer grains yielded more grain boundaries which hindered dislocation motion during tensile deformation. In addition, the dominant large SnSb phase precipitates which formed during static casting acted as notches and influenced the tensile cracking of the coating. 

### 3.3. Hardness Test

**Figure 8:**
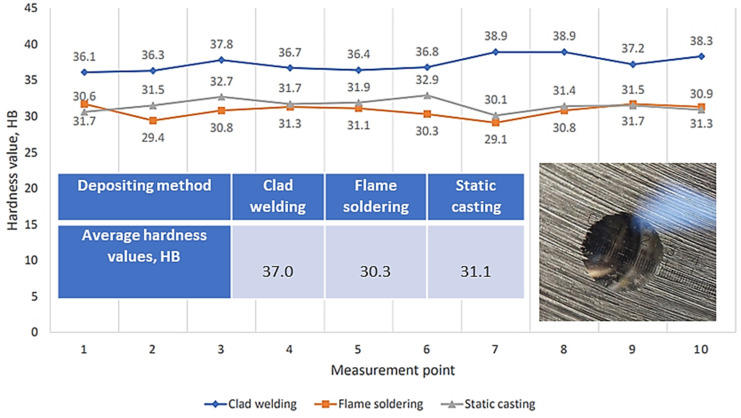
Hardness testing results.

The hardness of the coatings was tested using the Brinell method at a constant load of 1000 g according to ISO 6506-1:2014 [60]. A sintered carbide ball with a diameter of 5.0 mm was used to obtain the indents during the test. The average hardness results are summarized in Figure 8. 

To investigate the reason for the higher hardness in the coatings obtained using the clad welding technique, the chemical composition was analyzed with a spectrometer, focusing on the Sb and Cu contents. As microstructural studies have shown, Sb influences the formation of finer-grained structures and promotes the formation of solid intermetallic phases, such as Sn and Sb compounds, in the microstructure. Deposition of the coating using the clad welding method resulted in higher contents of Cu and Sb in the Babbitt, which increased the total volume fraction of Cu_6_Sn_5_ and SbSn precipitates, influencing the greater hardness of the Babbitt coating. The resulting fine precipitates were evenly distributed throughout the tin matrix. These formed phases contributed to the overall hardness of the Babbitt, which was confirmed by the hardness measurements.

In summary, the hardness test results of the three Babbitt alloy coating methods revealed clear differences. The coating fabricated using the clad welding method offered the highest hardness (with average hardness values of about 37.0 HB). The average hardness values of the coatings obtained with the flame soldering and static casting methods were about 30.3 HB and 31.1 HB, respectively (Figure 8). These average differences in hardness values were associated with differences in the microstructures and chemical compositional changes under each coating method. The higher surface hardness of the coating under operational conditions would make such a coating more resistant to intensive wear. Chemical composition analysis showed that the greatest amount of Sb (13.10%) and Cu (5.57%) remained in the Babbitt when using the clad welding method (Table 2).

### 3.4. Microstructure

**Figure 9:**
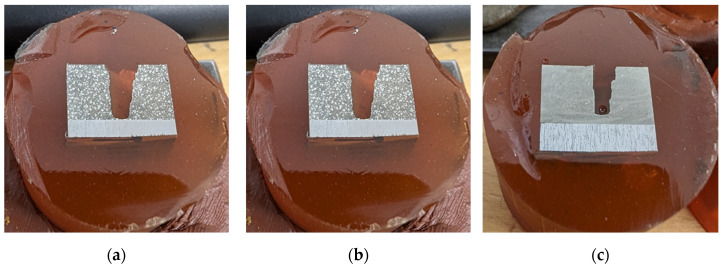
Microsections prepared for microstructural analysis: (**a**) static casting; (**b**) flame soldering; and (**c**) clad welding.

An analysis of the coating’s microstructure revealed information about its grain size, distribution, and morphology, which directly affected the mechanical strength, hardness, and other properties of the material. This analysis also helped identify any damage, defects, or other anomalies that could affect the performance of the deposited layer. We selected round “O-shaped” specimens for the microstructural analysis. The microsections (Figure 9) for the microstructural analysis were prepared using metallographic techniques and chemically etched with 4% nitric acid (HNO_3_) after polishing with a SMARTLAM 2.0 single-plate polishing machine (LAM PLAN S.A., Gaillard, France).

**Figure 10:**
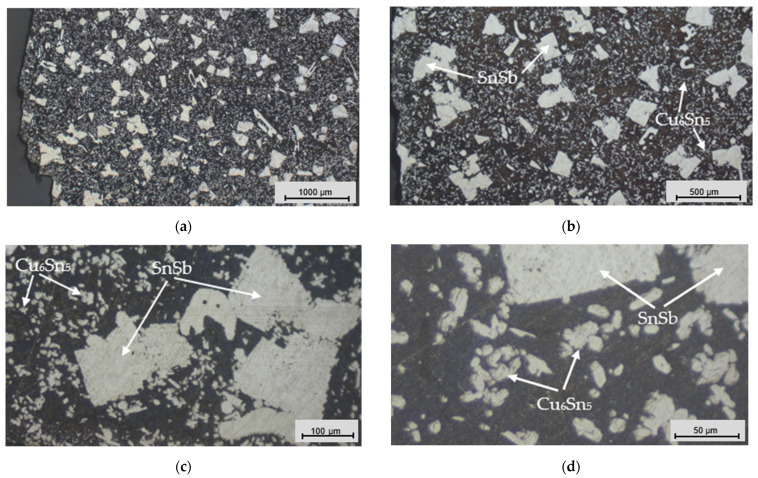
Microstructural images of the Babbitt alloy coating deposited via static casting: (**a**) magnification of 2.5×; (**b**) magnification of 5×; (**c**) magnification of 20×; and (**d**) magnification of 50×.

**Figure 11:**
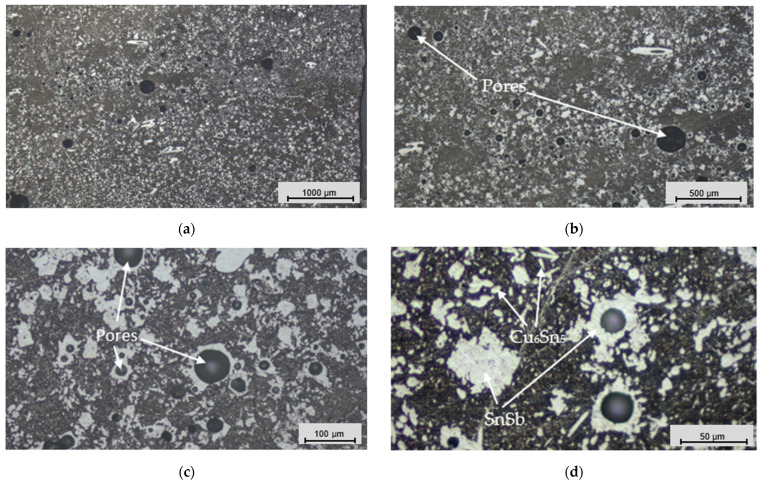
Images of the microstructures of the Babbitt alloy coating deposited via the flame soldering method: (**a**) magnification of 2.5×; (**b**) magnification of 5×; (**c**) magnification of 20×; and (**d**) magnification of 50×.

**Figure 12:**
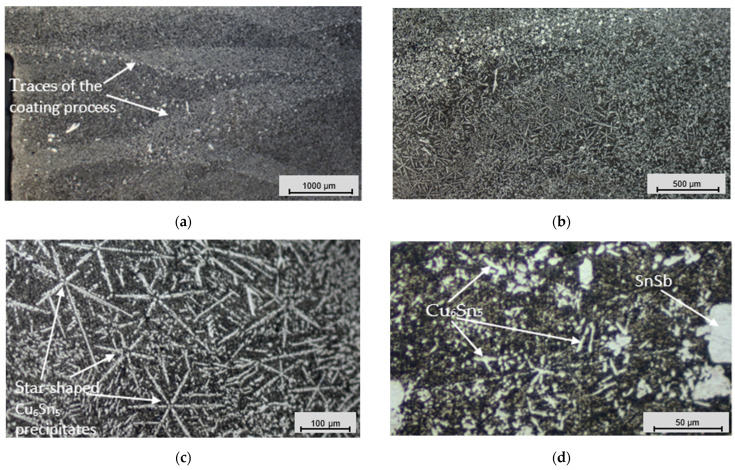
Microstructural images of the Babbitt alloy coating deposited via the clad welding method: (**a**) magnification of 2.5×; (**b**) magnification of 5×; (**c**) magnification of 20×; and (**d**) magnification of 50×.

Figure 10, Figure 11 and Figure 12 present trinocular microscope images of the microstructures of the coatings deposited via different casting methods.

Relatively large SnSb phase precipitates were clearly visible in the microstructure (Figure 10), next to which were small precipitates of the Cu_6_Sn_5_ phase. The microstructure of the statically cast Babbitt layer, characterized by dominant, large SnSb phase precipitates, had a significant influence on the coating properties. Incomplete homogenization of the alloy is one reason why alloy components such as Sn and Sb mix and melt unevenly during casting. Rapid solidification and non-uniform cooling rates during casting contributed to the formation of larger precipitates, which created a coarse-grained structure.

The microstructure of the Babbitt layer deposited via the flame soldering method presented a complex arrangement characterized by numerous pores and different precipitates (Figure 11). In the microstructure, larger SnSb phase precipitates surrounded by smaller Cu_6_Sn_5_ derivatives formed a heterogeneous network in the coating. However, the combined precipitates were not as widely distributed as in static casting, which improved the overall quality of the coating.

Pore formation affects the structural integrity and mechanical properties of the coating, an interesting aspect of which is the pore closure in SnSb compounds. This localization indicates a relationship between the formation of precipitates and the appearance of pores during the casting and solidification process. The microstructure confirmed the results of the mechanical tests, as the lower tensile strength here (Figure 7) was due to the relatively high porosity and the reduced density of the deposited layer’s structure.

Numerous star-shaped formations (asterisks) were observed in the microstructure of the Babbitt layer deposited via clad welding with a tungsten electrode in an argon gas environment, showing the dominance of Cu_6_Sn_5_ precipitates (Figure 12). These crystalline formations resulted from interactions between the electrode material and the molten Babbitt alloy. Due to the controlled solidification conditions, a fine microstructure was formed, which improved the mechanical properties and wear resistance of the coating [61,62,63]. Traces of the coating process were visible in the microstructure (Figure 12a), which appeared due to the electric arc. These traces provided a visual representation of the Babbitt’s material transfer path onto the surface of the base metal, demonstrating the ability to control the coating operation. In contrast to other casting methods, the microstructure under clad welding contained fewer large SnSb phase precipitates. The star-shaped formations, fine structure, complete absence of pores (Figure 12), and controlled casting process underscore the advantages of the clad welding method over other casting methods. These observations were also confirmed by the chemical composition (Table 2), tensile strength (Figure 7 and Table 3), and hardness tests (Figure 8), the results of which indicated the superiority of the clad casting method. The elimination of pores in the Babbitt coating under the clad welding method produced a coating with high adhesion strength to the substrate, as reflected in the reduced probability of surface damage.

The microstructural analysis explored the research results for different methods of Babbitt layer deposition. The outcomes highlighted the exceptionally fine structure of the Babbitt formed under the clad welding method, characterized by star-shaped Cu_6_Sn_5_ phase precipitates (Figure 12c). The flame soldering method was characterized by a relatively high porosity for the SnSb phase precipitates (Figure 11b,c), while the direct casting method was characterized by large SnSb phase precipitates which dominated the structure (Figure 10).

## 4. Conclusions

The research results yielded the following conclusions. The investigated Babbitt alloy coatings were characterized by a multiphase microstructure consisting of large precipitates of solid SnSb phases and numerous precipitates of Cu_6_Sn_5_ phases, which were both needle- and star-shaped. The matrix of deposited coatings was a solution of Sb and Cu in Sn. Our research showed that the coatings deposited via different methods differed in their microstructures, mainly due to the distribution and shapes of the CuSn and SnSb phase precipitates. In the coating deposited via static casting, the SnSb precipitates were relatively large and rhomboidal, resulting in a coarse-grained structure which reduced the mechanical properties of the coating compared with those of the coating deposited with the clad welding method, which was dominated by star-shaped Cu_6_Sn_5_ phase precipitates. This phenomenon was observed after performing tensile tests, in which the average ultimate tensile strengths of the specimen coatings fabricated via static casting and clad welding were 59 MPa and 83 MPa, respectively.

When casting using the clad welding method, we obtained more homogenous melted layers. In addition, the grain size of the intermetallic SnSb was more refined compared with that under static casting and flame soldering, yielding Babbitt with a greater hardness (37 HB versus 31.1 HB and 30.3 HB, respectively) due to grain refinement and homogenization of the microstructure. Overall, the presence of star-shaped Cu_6_Sn_5_ phase precipitates in the Babbitt layers had a critical effect on the material’s mechanical properties (hardness, plasticity, and tensile strength).

Analyses of the chemical composition showed that the quantity of chemical elements (5.57% Cu and 13.10% Sb) in the layer deposited using the clad welding method remained almost unchanged compared with the amounts in the original raw material (5.60% Cu and 13.40% Sb). Conversely, a decrease in these elements was clearly reflected in the layers deposited using the other two methods. When depositing via the flame soldering method, the Cu and Sb contents decreased to 3.69% and 12.70%, respectively. Under the direct casting method, these contents decreased to 3.73% and 12.40%, respectively.

The present research revealed that a higher amount of Sb yielded a coating with greater hardness. In addition, a higher amount of Cu increased the tensile strength of the coating. However, excessive contents of these two elements are known to reduce the ductility of the coating, and this it is extremely important to control the desired concentrations of Sb and Cu. The results of the experiments showed that the chemical composition of the coating changed little from that of the Babbitt alloy when using the clad welding method to deposit the alloy under a protective argon gas environment.

## Figures and Tables

**Table 1:** Chemical composition of the Babbitt alloy remelted rods.

Chemical Element	Fe	Al	Cu	As	Pb	Zn	Sb	Bi	Sn
Amount (wt.%)	0.090	0.002	5.60	0.007	0.094	0.006	13.40	0.001	Bal.

**Table 2:** Chemical composition analysis data.

Deposition Method	Percentage of Chemical Elements in the Composition (wt.%)							
Static casting	Sn	Sb	As	Bi	Pb	Cu	Fe	Ni
	Balance	12.40	0.007	0.001	0.034	3.73	0.090	0.001
	Al	Zn	Cd	Ag	Co	In		
	0.002	0.006	0.001	0.001	0.003	0.004		
Flame soldering	Sn	Sb	As	Bi	Pb	Cu	Fe	Ni
	Balance	12.70	0.007	0.001	0.093	3.69	0.090	0.001
	Al	Zn	Cd	Ag	Co	In		
	0.002	0.006	0.001	0.001	0.002	0.006		
Clad welding	Sn	Sb	As	Bi	Pb	Cu	Fe	Ni
	Balance	13.10	0.007	0.001	0.051	5.57	0.090	0.001
	Al	Zn	Cd	Ag	Co	In		
	0.002	0.006	0.001	0.001	0.003	0.004		

**Table 3:** Data from the tensile strength testing experiment.

Description of the Specimen		Max. Load F_max_ (kN)	Ultimate Tensile Strength R_m_ (MPa) (N/mm^2^)	The Location of the Failure
Coating Method	No. of the Specimen			
Static casting (SC)	SC1	8.38	69.00	Bonding point of the Babbitt to the base metal
	SC2	5.58	46.00	Bonding point of the Babbitt to the base metal
	SC3	9.37	78.00	Bonding point of the Babbitt to the base metal
	SC4	5.22	44.00	Bonding point of the Babbitt to the base metal
	SC5	7.04	58.00	Bonding point of the Babbitt to the base metal
∆59.00				
Flame soldering (FS)	FS1	6.97	58.00	Bonding point of the Babbitt to the base metal
	FS2	6.78	57.00	Babbitt coating
	FS3	9.30	77.00	Babbitt coating
	FS4	10.16	84.00	Babbitt coating
	FS5	9.46	78.00	Bonding point of the Babbitt to the base metal
∆71.00				
Clad welding (CW)	CW1	12.16	102.00	Bonding point of the Babbitt to the base metal
	CW2	8.04	67.00	Bonding point of the Babbitt to the base metal
	CW3	9.71	80.00	Bonding point of the Babbitt to the base metal
	CW4	10.66	88.00	Babbitt coating
	CW5	9.42	79.00	Babbitt coating
∆83.00				

## Data Availability

Data are contained within the article.
